# Association of integrin beta1 and c-MET in mediating EGFR TKI gefitinib resistance in non-small cell lung cancer

**DOI:** 10.1186/1475-2867-13-15

**Published:** 2013-02-13

**Authors:** Lixia Ju, Caicun Zhou

**Affiliations:** 1 Department of Traditional Chinese Medicine, Shanghai Pulmonary Hospital, Tongji University, Medical School, Shanghai, 200433, China; 2 Cancer Institute, Department of Oncology, Shanghai Pulmonary Hospital, Tongji University, Medical School, 507 Zhengmin Road, Shanghai, 200433, China

**Keywords:** Non-small cell lung cancer, Integrin beta1, c-MET, EGFR TKI, Resistance

## Abstract

Although some patients are initially sensitive to epidermal growth factor receptor tyrosine kinase inhibitors (EGFR TKIs), resistance invariably develops. Therefore, it’s very important to study the molecular mechanism of this resistance. In our previous study we found that integrin beta1 can induce EGFR TKIs resistance in non-small cell lung cancer (NSCLC) cells. Here we analyzed the association of integrin beta1 and c-MET that is a recognized mechanism of EGFR TKIs resistance in NSCLC to demonstrate the mechanism of integrin beta1 related EGFR TKIs resistance. We found that the ligands of integrin beta1 and c-MET could synergistically promote cell proliferation and their inhibitors could synergistically improve the sensitivity to gfitinib, increase apoptosis, and inhibit the downstream signal transduction: focal adhesion kinase (FAK) and AKT. On the other hand, ligand-dependent activation of integrin beta1 could induce EGFR TKIs resistance through activating c-MET and its downstream signals. Thus, it can be concluded that there is crosstalk between integrin beta1 and c-MET and integrin beta1 mediates EGFR TKI resistance associating with c-MET signaling pathway in non-small cell lung cancer.

## Introduction

The EGFR inhibitor gefitinib has been used as a single agent in NSCLC, but overall, the resistance remains a major problem clinicians encountered. Our previous result has shown that integrin beta1 overexpression associates with EGFR TKI resistance in PC9/AB2 cells [1]. In this study we further investigated the mechanism of integrin beta1-related EGFR TKI resistance.

Integrins and are formed by α and β integrin subunits. There are at least 24 known heterodimers formed by 18 α and eight β subunits. Natural integrin ligands include important components of the extracellular matrix (ECM). Beta1 subunit of integrin is an adhesion molecule involved in cell survival and cancer resistance to radiotherapy and chemotherapy [2-4], sharing common downstream signaling elements with EGFR, such as the phosphatidylinositol 3-kinase/AKT and extracellular signal-regulated kinase-1/2 (ERK1/2) pathways [5-9].

The c-MET receptor is a 190-kD disulfide linked α-β heterodimer [10]. and expressed in 60%–80% of NSCLC [11]. Unlike EGFR, the only known natural ligand for c-MET is hepatocyte growth factor (HGF, also known as scatter factor). Activation of c-MET can lead to proliferation, increased survival, altered motility, enhanced invasion into extracellular matrix, and more rapid formation of tubules [12]. On activation by autophosphorylation, c-MET can activate its multiple downstream signal transduction intermediates. Novel small molecule inhibitors of c-MET, SU11274 [13] and PHA-665752 [14] have shown to inhibit the phosphorylation of c-MET and the proliferation of cells in vitro.

In recent years, c-MET also has been found to be an independent biomarker of EGFR TKI resistance and about 21% acquired EGFR TKI resistance is caused by overexpression of c-MET [15]. Another research found that through promoting MET-integrin association, HGF-FN and HGF-VN complexes coordinated and enhanced endothelial cell migration through activation of the PI-3 kinase pathway involving a Ras-dependent mechanism [16]. There is also an important crosstalk between c-MET and the integrin beta1 in mast cell: stimulation through c-MET and the α2β1 integrin resulted in crosstalk between the two receptors, resulting in the activation of the mast cell leading to release of the pro-inflammatory cytokine, IL-6 [17]. Therefore, the crosstalk between integrin beta1 and c-MET may be also related with EGFR TKI resistance.

In present study, we investigated the relationship between integrin beta1 and c-MET in EGFR TKI resistance to explore the mechanism of EGFR TKI resistance in non-small cell lung cancer.

## Materials and methods

### Ethics approval

All experiments were performed with the approved of the Tongji University Institutional Care and Use Committee (IACUC).

### Reagents and antibodies

Human phospho-ERK antibody and human phospho-FAK antibody were purchased from Animal BioWorld Technology (Dublin, OH); human c-MET antibody, human phospho-AKT antibody, human phospho-c-MET antibody and human phospho-EGFR antibody were purchased from Cell Signaling Technology (Beverly, MA); β-actin antibody was purchased from ABGENT (San Diego, CA); human integrin beta1/CD29 antibody was purchased from R&D Systems (Minneapolis, MN); IRDyeTM 800 Conjugated Affinity Purified Anti-mouse/rabbit Antibody was purchased from Rockland (Gilbertsville, PA).

### Cell lines and cell culture

Human NSCLC cell line PC9 (harboring EGFR exon 19 deletion) was provided by Cancer Institute of Medical School, Tongji University, China [the original PC-9 cells were purchased from Immuno-Biological Laboratories (Takasaki, Gunma, Japan). The gefitinib-resistant NSCLC subline PC9/AB2 was induced from PC9 cells according to the method in the literature [18] and was continuously subcultured with 2 μmol/L of gefitinib for additional 6 months. The resistance of PC9/AB2 cells to gefitinib has been proved to maintain for at least one year in the medium without gefitinib and there is no T790M in PC9/AB2 [1]. PC9/AB2 cells were stablely transfected with integrin beta1-siRNA plasmid and scrambled siRNA plasmid and were named by AB2/17-2 and AB2/N respectively; The integrin beta1 cDNA plasmid and the vacant vector were stablely transfected into PC9 cells and were named by PC9/D6, PC9/PCD respectively [1]. All these cells were cultured at 37°C with 5% CO2 in Dulbecco’s modified Eagle’s medium (DMEM) supplemented with 10% fetal bovine serum (FBS), 100 U/ml penicillin and 100 mg/ml streptomycin.

### Western blot assay

Cells were washed twice with ice-cold PBS and lysed in 0.1 ml of lysis buffer on ice for 30 min. Insoluble debris was removed by centrifuging at 13,000 rpm for 15 min at 4°C. Electrophoresis and blotting procedures were done according to methods described previously. Primary antibodies against human integrin beta1/CD29, human c-MET antibody, human phospho-EGFR and phospho-AKT, phospho-ERK1/2 and phospho-FAK were used according to the manufacturer’s instructions. Blotting quantification was done with an Odyssey® Infrared Imaging system (LI-COR, USA).

### Fibronectin (FN) stimulate growth

96-well plates were coated with or without fibronectin (FN) 20 μg/ml 100 μl. PC9, PC9/AB2 (5000 /well) were plated in 96-well plates. After 24 h, the wells were treated with or without HGF 50 ng/ml, or treated with both of them in medium with 10% FBS. After 24 h, viable cells were detected by MTT assays and cell viability was plotted as a mean ± SD of three independent experiments. Percentage of cell viability was determined relative to the control that had no additional growth factors [19].

### Cell proliferation assay

The cells (5 × 10^3^/well) were seeded into 96-well plate in quadruplicate and were exposed to various concentrations of gefitinib. After 72 hours, 20 μl of 3-(4,5-diMEThylthiazol-2-yl)-2,5-diphenyltetrazolium bromide (MTT) solution (5 mg/ml) was added to each well and incubated. After 4 hours, crystalline formation was dissolved with Dimethyl sulfoxide (DMSO) and the absorbance at 530 nm was read using the microplate-reader for ELISA MK-2 Labsystems Dragon. The IC50 was defined as the concentration needed for a 50% reduction of the absorbance based on the survival curves. Percent survival was calculated as: (mean absorbance of the replicate wells containing drugs^_^mean absorbance of the replicate background wells) / (mean absorbance of the replicate drug-free wells^_^mean absorbance of the replicate background wells). The test was performed independently 3 times. All results were derived from quadruplicate experiments yielding almost similar results.

### Apoptosis assay

Transferase-mediated deoxyuridine triphosphate nick-end labeling (TUNEL) Kit (Promega, USA) were used for apoptosis assay. In TUNEL assay, cells were seeded in 24-well plates and exposed to gefitinib (5 or 15 μmol/L) for another 48 h. Then, apoptosis was assessed by the TUNEL assay kit (GENMED, China) following the manufacturer’s protocol. Apoptotic index (AI) (%) was calculated by the formula: positive staining cells/tumor cells number × 100%.

### Statistical analyses

Values were expressed as mean ± SD. Statistical analysis was done by independent-samples t test. Differences were considered to be statistically significant if P < 0.05.

## Results

### Integrin beta1 and c-MET co-expressed in PC9 and PC9/AB2 cell lines

**Figure 1:**
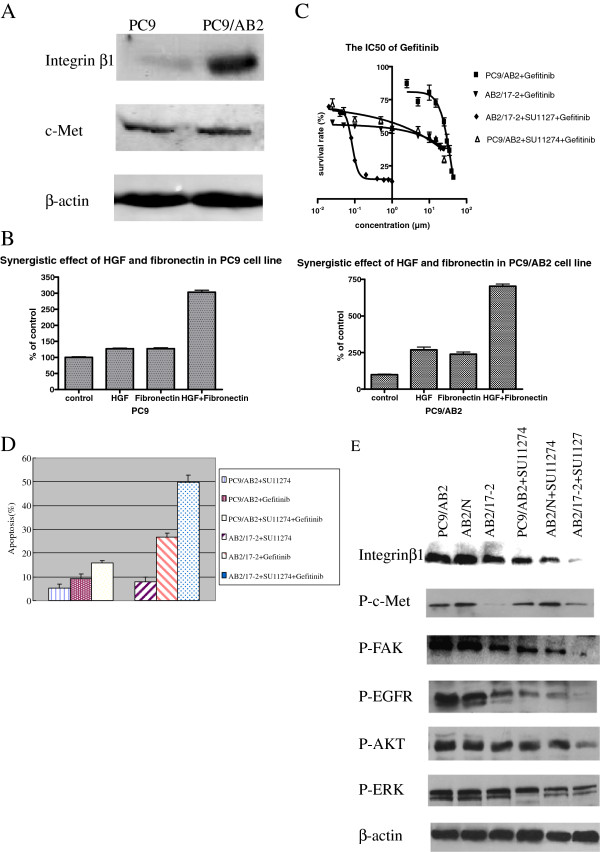
**The crosstalk between integrin beta1 and c**-**MET.A** Western blot analysis showed that integrin beta1 and c-Met co-expressed in PC9 and PC9/AB2 cells. **B** MTT analysis of proliferative effect of PC9 and PC9/AB2 cells treated with FN and HGF alone or in combination showed a synergistic proliferative effect when FN and HGF combined. **C** MTT analysis showed that combination of integrin beta1-target siRNA and c-MET kinase inhibitor SU11274 synergized to inhibit proliferation of PC9/AB2 NSCLC cell line. **D** TUNEL analysis showed that inhibition of c-Met and integrin beta1 concurrently induced apoptosis synergistically. **E** Western blot analysis showed that phosphorylation of c-Met, EGFR, FAK and AKT were reduced synergistically when c-Met and integrin beta1 were inhibited in PC9/AB2 cells. *G means treatment of gefitinib and P means phosphorylation.

The expression of integrin beta1 and c-MET were determined by western blot. Integrin beta1 and c-MET were both expressed in the PC9 and PC9/AB2. Integrin beta1 expression was higher in PC9/AB2 than in PC9, and c-MET expression was comparable in the two cell lines (Figure 1A).

### Fibronectin (FN) and HGF effect on the growth of PC9 and PC9/AB2 cell lines

FN is the ligand of integrin beta1 and HGF is the ligand of c-MET. The effects of FN and HGF on cell growth were studied by treating cells with 20 μg/ml FN or 50 ng/ml of HGF alone or in combination, and cell proliferation was determined by MTT as aforementioned. In PC9 and PC9/AB2 cells, a synergistic proliferative effect was observed with a combination of FN and HGF (Figure 1B).

A synergistic effect can be defined as an effect that is more than the additive effect of FN and HGF alone. Combination Index (CI) = (D) 1/ (D m) 1 + (D) 2 /(D m) 2 . Where (D) 1 and (D) 2 are the doses of chemicals 1 and 2 that in combination produce some specified effect (i. e. 50% inhibition of luminescence) and (D m) 1 and (D m) 2 are the doses of the chemicals that when applied singly also have the same effect (50% inhibition of luminescence).

These results indicated that there was a synergistic proliferative effect of both ligands combined. Maybe because the level of integrin beta1 in PC9/AB2 cells is higher than that in PC9 cells, the synergistic effect of HGF and FN in PC9/AB2 cells is more evident than in PC9 cells. These results could be due to potential crosstalk between integrin beta1 and c-MET signaling pathways.

### Combination of integrin beta1-target siRNA and c-MET kinase inhibitor SU11274 synergized to inhibit proliferation of PC9/AB2 NSCLC cell line

Since the synergistic proliferative effect of HGF and FN has been observed, we want to know whether the synergistic inhibition to cell growth can be found when we combined integrin beta1 siRNA with c-MET kinase inhibitor SU11274. The IC50 for each treatment was determined by MTT assay. The results shown that the IC50 for gefitinib alone and the combination of SU11274 and gefitinib were (24.2 ± 5.45) μmol/L and (7.34 ± 2.84) μmol/L in PC9/AB2 cells, respectively. Interestingly, a synergistic effect of gefitinib on inhibition of cell proliferation was seen in the presence of same dose of SU11274 in integrin beta1-inhibited AB2/17-2 cells. The IC50 for gefitinib alone in AB2/17-2 cells was (1.9 ± 1.28) μmol/L, however the IC50 was (0.26 ± 0.15) μmol/L in the presence of same dose of SU11274 (Figure 1C). So, to inhibit the cells growth by 50%, we need 30% or 8% of original gefitinib concentration respectively in presence of SU11274 or integrin beta1-siRNA only. But we need only 1% of original gefitinib concentration when SU11274 and integrin beta1-target siRNA were combined. It suggested that combined inhibition of integrin beta1 and c-MET could improve effect of gefitinib in PC9/AB2 NSCLC cell line synergistically.

### Combination of integrin beta1-target siRNA and c-MET kinase inhibitor SU11274 induced apoptosis in a synergistic fashion

TUNEL assay was performed to examine apoptosis. As shown in Figure 1D, the apoptosis rates of PC9/AB2 cells treated with SU11274 or gefitinib alone or in combination were (5.38 ± 1.94)%, (9.33 ± 0.98)%, and (15.48 ± 2.32)% respectively. And the apoptosis rates of integrin beta1-inhibited AB2/17-2 cells treated with SU11274 or gefitinib alone or in combination were (7.8 ± 1.56)%, (26.68 ± 3.10)%, and (49.72 ± 6.82)% respectively. It suggested that the combination of integrin beta1-target siRNA and SU11274 could increase apoptosis induced by gefitinib in PC9/AB2 cell line in a synergistic fashion.

### Combination of integrin beta1-target siRNA and c-MET kinase inhibitor SU11274 reduced phosphorylation of EGFR and its downstream signals synergistically

After 30min of treatment with EGF, we investigated the phosphorylation level of EGFR and several of its downstream signaling intermediates (Figure 1E). The synergistic reduction of phosphorylation levels were observed in EGFR, AKT and FAK. Phosphorylation of ERK decreased significantly with c-MET inhibition but not with integrin beta1 inhibition. The results indicated that there was a crosstalk between c-MET and integrin beta1, and activation of AKT and FAK were very important for this crosstalk.

### Activation of integrin beta1 by FN increased gefitinib resistance

**Figure 2:**
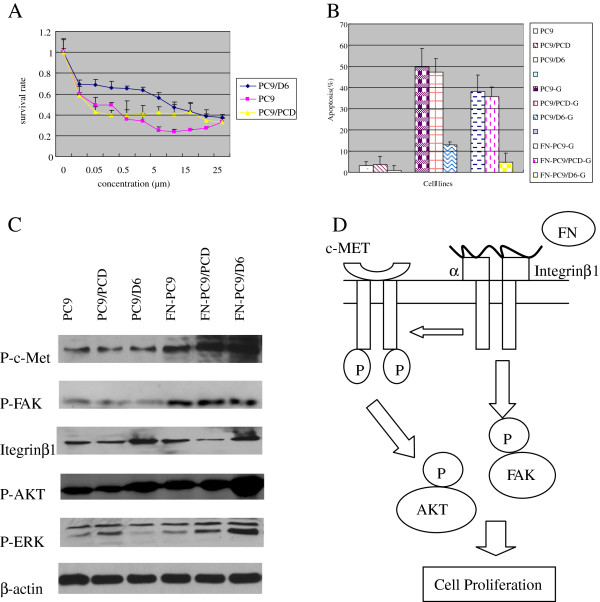
**Overexpression and activation of integrin beta1 induce gefitinib resistance via c**-**MET signaling pathway.A** MTT analysis showed that activation of integrin beta1 by FN could induce gefitinib resistance. **B** TUNEL analysis showed that the apoptosis of PC9/D6 cells were decreased markedly after treated with FN. **C** Western blot analysis showed that ligand-dependent activation of integrin beta1 could increase phosphorylation of c-Met, FAK, and AKT in PC9/D6 cells. **D** Overexpression of integrin beta1 induces EGFR TKI resistance through increasing phosphorylation of c-MET and its downstream signals: FAK, AKT.

In our previous research, we found that FN could increase cell adhesion^1^, so we investigated whether or not activation of integrin beta1 by FN could improve survival and increase gefitinib resistance. The IC50 of gefitinib in PC9 and PC9/D6 cells were (0.042 ± 0.01) μmol/L and (9.26 ± 1.20) μmol/L respectively [1]. The IC50 of gefitinib were (0.08 ± 0.03) μmol/L and (17.50 ± 4.15) μmol/L in PC9 and PC9/D6 cells when co-treated with FN, respectively (Figure 2A). These data suggested that activation of integrin beta1 by FN could induce gefitinib resistance.

### Activation of integrin beta1 by FN decreased gefitinib-induced apoptosis

To further confirm that the activation of integrin beta1 by FN have some effect on EGFR TKI resistance, we detected the apoptosis of PC9, PC9/D6 cells treated with gefitinib with or without FN. The apoptosis rates were (3.09 ± 1.80) %, (0.95 ± 2.13) % in PC9 and PC9/D6 cells, respectively. Their apoptosis rates were (49.75 ± 8.60) %, (12.87 ± 1.53) % when treated with gefitinib. However, they were (38.09 ± 7.84) %, (4.20 ± 4.29) % when treated with FN and gefitinib, respectively (Figure 2B). The apoptosis were both decreased after treated with FN, but that of integrin beta1-overexpressed PC9/D6 cells descended markedly.

### Ligand-dependent activation of integrin beta1 induced c-MET and its downstream signals activation

After activation of integrin beta1 by FN, phosphorylation of c-MET, FAK, AKT, and ERK were all increased significantly. It suggested that ligand-dependent activation of integrin beta1 induced c-MET activation and phosphorylation of FAK, AKT, and ERK were related to this effect. So we concluded that ligand-dependent activation of integrin beta1 could induce EGFR TKI resistance through increasing phosphorylation of c-MET and of its downstream signaling pathways: FAK, AKT and ERK (Figure 2C).

## Discussion

In this work, we demonstrate that there is crosstalk between integrin beta1 and c-MET and this crosstalk regulates EGFR TKIs resistance in NSCLC. We provide evidence that integrin beta1/MET crosstalk is a key factor of EGFR TKIs resistance, thus rendering integrin beta1/c-MET a suitable double-target for adjuvant therapy in combination with anti-EGFR agents currently used in clinic.

Although the patients with mutant EGFR display dramatic response to EGFR TKIs, duration of response is typically only 9 to 10 months and then most patients eventually acquire resistance to the agents [20,21], leading to treatment failure. Mechanisms for acquired resistance to EGFR TKIs have been widely studied. T790M mutation and c-MET gene amplification have been found to be related to acquired resistance to EGFR TKIs in NSCLC [22-24]. Except for the above two mechanisms, others that account for the remaining about 30% of acquired resistance are still unclear. Some papers showed that integrin beta1 signaling has been implicated in the progression and metastasis of various cancers, and shown to facilitate resistance to radiation therapy [25] and drug resistance [26]. Moreover, our previous research had confirmed that integrin beta1 was responsible for EGFR TKI resistance.

Integrin beta1, that associates with the adhesion and migration capability of tumor cells and has a key role in the growth and metastasis of tumors, is an important molecular of the adhesion-mediated drug resistance [27-29]. The FN receptor (α5β1-integrin) binds to fibronectin to anchor cells and activates non receptor tyrosine kinases, FAK and Src, which play an important role in tumorigenesis by promoting the proliferation and invasion of cancer and endothelial cells [30,31]. In our previous research, we have established the cell lines with stable down- and up- expression of integrin beta1 by transfecting siRNA or integrin beta1 cDNA plasmid into PC9/AB2 cells or PC9 cells, respectively. After down-regulation of integrin beta1, PC9/AB2 cells partially restored sensitivity to gefitinib while up-regulation of integrin beta1 led to resistance of PC9 cells to gefitinib. Expression level of integrin beta1 was negatively correlated with gefitinib sensitivity in these two cell lines. These data identified that integrin beta1 is an important factor of EGFR TKIs resistance. Morello also found that the integrin beta1-silenced cells showed a defective activation of the EGFR signaling cascade, leading to decreased in vitro proliferation, enhanced sensitivity to gefitinib, impaired migration and invasive behavior [5].

Our results showed that both integrin beta1 and c-MET were expressed in these cell lines, and their ligands can enhance cell proliferation synergistically. Importantly, inhibition of both receptors led to growth inhibition and apoptosis, and down-regulation of phosphorylation of molecules in their downstream signal transduction (such as the AKT, FAK pathways) in a synergistic fashion. Ligand-dependent activation of integrin beta1 induced c-MET and it’s downstream signals activation (FAK, and AKT). Mitra et al. also reported that inhibition of α(5)β(1)-integrin decreased the phosphorylation of c-Met, FAK and Src, both in vitro and in vivo. Activation of c-Met by its ligand, HGF/SF, or overexpression of a constitutively active FAK in HeyA8 cells could overcome the effect of α(5)β(1)-integrin inhibition on tumor cell invasion, indicating that α(5)β(1)-integrin is upstream of c-Met, Src and FAK. Inhibition of α(5)β(1)-integrin on cancer cells in two xenograft models of ovarian cancer metastasis resulted in a significant decrease of tumor burden, which was independent of the effect of α(5)β(1)-integrin on angiogenesis [32]. These data shows that there is a crosstalk between integrin beta1 and c-MET signaling pathways, and it reaches consensus with Beviglia’s results that the two signaling pathways, integrin/ECM and c-MET/HGF, cooperate synergistically to induce FAK activation in an adhesion-dependent manner, leading to enhanced cell adhesion and motility [33].

In conclusion, we identified that the crosstalk between integrin beta1 and c-MET via AKT and FAK signaling pathways is very important in EGFR TKI resistance. Our findings identified a new molecular mechanism of EGFR TKIs resistance, which will provide an effective therapeutic intervention of EGFR TKIs resistance.

## Competing interests

None of the authors of this study has a conflict of interest statement.

## Authors’ contributions

ZC contributed to concept development and study design. JL performed the experiments and draft the manuscript. Both authors read and approved the final manuscript.
